# Hepatobiliary Iminodiacetic Acid (HIDA) Scan in the Evaluation of Biliary Atresia: A Retrospective Cohort Study

**DOI:** 10.7759/cureus.83482

**Published:** 2025-05-04

**Authors:** Fatma M Al Hajri, Badriya Al Suqri, Khalid Al Busaidi, Athari Al-Obaidani

**Affiliations:** 1 Nuclear Medicine, Royal Hospital, Muscat, OMN; 2 Radiology, Oman Medical Specialty Board, Muscat, OMN

**Keywords:** biliary atresia, cholestatic jaundice, liver biopsy, neonatal hepatitis, neonatal jaundice

## Abstract

Objectives

This study aims to evaluate the accuracy of hepatobiliary iminodiacetic acid (HIDA) scan in infants, based on examinations performed in the nuclear medicine department at the Royal Hospital. The scan results will be compared to clinical outcomes following liver biopsy, considering ultrasound findings and liver enzyme results. The findings will be analyzed alongside published data through a literature review. This study is expected to contribute to the standardization of imaging protocols, support a multidisciplinary diagnostic approach, and ultimately enhance affected infants' diagnosis, prognosis, and quality of life.

Methods

The Royal Hospital Research Committee granted official ethical approval for this research. The HIDA scans done from 2015 to the end of 2023 were retrospectively evaluated. The data was collected by accessing medical records, clinical files, and imaging in picture archiving and communications systems (PACS). Diagnostic accuracy of HIDA scans in detecting problems with the gallbladder was measured using the sensitivity, specificity, positive predictive value, and negative predictive value (NPV). HIDA scan results were compared with the histopathology findings and the clinical follow-ups. A P-value less than 0.05 was considered statistically significant.

Results

A total of 53 infant patients were included in this cohort retrospective analysis. The most common presentation was conjugated hyperbilirubinemia, observed in 88.7% of cases, with a mean conjugated bilirubin level of 115.3 µmol/L. HIDA scan results were abnormal in 33 patients (62.3%) and normal in 20 patients (37.7%). Based on combined histopathological findings and long-term clinical outcomes, 21 patients were confirmed to have biliary atresia (BA), while 32 were negative. The HIDA scan correctly identified 20 of the 21 actual cases. There were eight false-positive results in which the HIDA scan suggested BA, but patients improved without intervention, most later diagnosed with neonatal hepatitis. Only one case was a false negative.

Conclusion

The results align with the reported sensitivity and specificity in the literature. This shows that while the HIDA scan is highly sensitive and effective in detecting BA, it has moderate specificity. Therefore, histopathological evaluation remains essential for confirming the diagnosis. The high NPV makes the HIDA scan very helpful in ruling out the diagnosis of BA, hence avoiding further invasive investigations in patients with a negative HIDA result.

## Introduction

Biliary atresia (BA) is a serious infant condition, affecting the biliary tree and resulting in neonatal cholestasis and eventually leading to progressive liver injury [1].

Although BA is considered the most frequently recognized condition that causes obstructive jaundice in the first three months of life [2], it must be differentiated from other major causes of neonatal cholestasis, mainly neonatal hepatitis (NH). These two conditions have entirely different treatment plans and prognoses [3]. While BA is regarded as a surgical emergency, NH warrants medical treatment; hence, accurate differentiation between the two diagnoses is significant [3].

Guidelines have been established for evaluating cholestatic jaundice (including careful history, thorough physical examination, and fractionation of serum bilirubin, as well as imaging modalities), and they are considered highly beneficial in reaching an accurate diagnosis [2]. Despite that, BA remains a challenging disease condition that can still result in unfavorable outcomes [4].

Hepatobiliary iminodiacetic acid (HIDA) scintigraphy is a noninvasive functional imaging modality used to evaluate the hepatobiliary system [5]. It is considered a handy diagnostic tool in the workup of neonatal cholestasis [6]. 

Many studies recommend HIDA scintigraphy for the primary evaluation of jaundiced patients and as a screening test for BA, as it offers a safe, simple procedure free from complications [7]. Some authors recommend semi-quantitative parameters in HIDA scintigraphy (e.g., using kidney-to-liver ratio; KLR) to overcome the challenges in reporting such cases and possibly improve the accuracy of HIDA scintigraphy in diagnosing BA [3].

Some studies, however, have thought that although HIDA scintigraphy is considered sensitive in detecting biliary obstruction, it has low specificity (as it may not be able to distinguish BA from failure of bile excretion). Therefore, they consider this option harmful as it may delay the diagnosis and treatment [1]. The accuracy of HIDA scintigraphy in diagnosing BA can be affected by many factors, such as prematurity, age at testing, and the severity of cholestasis during examination. Hence, more evaluations and studies are suggested to recognize these factors, which can consequently improve the accuracy of HIDA scintigraphy [8]. However, for the best outcome, it is not recommended to delay the decision of surgical exploration in equivocal HIDA cases [8].

Surgical exploration with intraoperative cholangiography is usually required to confirm the absence of a patent biliary tree and establish the diagnosis of BA [9]. Pediatric gastroenterologists, however, prefer to perform percutaneous liver biopsy before surgery if a precise diagnosis is not yet established [9].

Surgical intervention in cases of BA, called Kasai portoenterostomy, aims to restore bile flow and reduce hepatic fibrosis and cirrhosis. If fibrosis or cirrhosis occurs, liver transplantation may be required in infancy. The surgery must be prompted to increase the likelihood of procedure success [1]. After Kasai portoenterostomy, follow-up with the serum bilirubin level remains the most accurate clinical predictor of native liver survival [4].

The prognosis in cases of BA depends on early detection and treatment, which warrants prompt surgery. HIDA scintigraphy can, therefore, play a pivotal role in the workup of neonatal jaundice, detecting biliary obstruction that needs urgent surgical intervention [5].

## Materials and methods

This retrospective cohort study included all patients who underwent HIDA scintigraphy to evaluate BA at the nuclear medicine department of the Royal Hospital between January 1, 2015, and December 31, 2023. Patients who defaulted on follow-up were excluded from the analysis. Ethical approval was obtained from the Royal Hospital Research Committee before data collection (HIDA scan in neonatal cholestasis, tertiary hospital experience, SRC#26590).

Patients were selected based on clinical suspicion of BA, and data were retrieved from medical records, imaging archives, and clinical files. Inclusion criteria included neonates and infants referred for suspected BA based on clinical findings, elevated liver enzyme levels, and ultrasound abnormalities. Patients were excluded if they did not have complete imaging studies or were lost to follow-up. HIDA scintigraphy was performed using a dual-head gamma camera with a wide field of view. The radiotracer 99mTc-labeled HIDA was administered at a dose determined according to the European Association of Nuclear Medicine (EANM) Dosage Card (Version 5.7.2016), with a minimum recommended dose of 30 megabecquerel (MBq).

Dynamic imaging was initiated immediately after radiotracer injection with the following parameters: matrix size of 128 × 128, zoom of 1.45, camera preset at 99mTc, and detector one positioned in a head-out, supine orientation to capture anterior views continuously for 60 minutes.

Static images were acquired at 2, 4, and 24 hours post-injection. Imaging parameters were adjusted to a matrix size of 256 × 256 and a zoom of 1.23. The camera preset remained at 99mTc, and detector one was used in the same head-out, supine orientation. Each static image acquisition targeted 500 kilo counts (kcts) over approximately two minutes, focusing on the anterior view only.

HIDA scan findings were analyzed in correlation with histopathology results whenever available and long-term clinical follow-up data (ranging from one to six years). Diagnostic accuracy was determined through sensitivity, specificity, positive predictive value (PPV), and negative predictive value (NPV). A comparative analysis was performed using the existing literature to assess the hospital’s diagnostic performance relative to international standards.

Statistical analysis

Continuous variables were presented as mean and standard deviation, whereas categorical variables were presented as frequency and percentage. Comparison of means between two groups was performed using the independent samples t-test. Association between two categorical variables was tested using the chi-square test (Fisher’s exact/Likelihood ratio). Diagnostic accuracy of the HIDA scan in detecting problems with the gallbladder was measured using the sensitivity, specificity, PPV, and NPV. HIDA scan results were compared with the histopathology findings and the clinical follow-ups. A P-value less than 0.05 was considered statistically significant. Analysis was done using MedCalc version 22 (MedCalc Software Ltd., Ostend, Belgium) and IBM SPSS Statistics for Windows, Version 29 (Released 2023; IBM Corp., Armonk, New York, United States).

## Results

The demographic characteristics of the study cohort, categorized by HIDA scan results, are detailed in Table 1. The study included 53 patients, with a mean age of 45.1 days (SD: 21.0 days) at the time of HIDA imaging. Patients with a positive HIDA scan were slightly younger, with a mean age of 42.3 days (SD: 17.6 days), compared to those with a negative HIDA scan, who had a mean age of 48.2 days (SD: 24.2 days). Regarding gender distribution, 71.7% (n = 38) of the total cohort were male, while 28.3% (n = 15) were female. Among patients with a positive HIDA scan, 64.3% (n = 18) were male, and 35.7% (n = 10) were female. Conversely, 80.0% (n = 20) were male in the negative HIDA scan group, and 20.0% (n = 5) were female.

**Table 1:** Demographic Data for the Subjects. HIDA scan: Hepatobiliary iminodiacetic acid scan

Demographic	All Patients	Positive HIDA scan	Negative HIDA scan
Age (days), mean (SD)	45.1 (21.0)	42.3 (17.6)	48.2 (24.2)
Male (%)	38 (71.7%)	18 (64.3%)	20 (80.0%)
Female (%)	15 (28.3%)	10 (35.7%)	5 (20.0%)

Neonatal jaundice was the most common presentation in this cohort of suspected BA, with nearly all patients exhibiting conjugated hyperbilirubinemia. Among 47 patients (88.7%), conjugated bilirubin levels had a mean of 115.38 µmol/L (SD: 62.97) and a median of 111 µmol/L, ranging from 13 to 310 µmol/L. Gamma-glutamyl transferase (GGT) levels, analyzed in 46 patients (86.8%), had a mean of 405.81 IU/L (SD: 430.65) and a median of 234 IU/L, with values spanning 31.37 to 2133 IU/L. Similarly, ALP levels, measured in 51 patients (96.2%), had a mean of 626.48 IU/L (SD: 309.68) and a median of 595 IU/L, ranging from 21 to 1713 IU/L. The broad variation in conjugated bilirubin, GGT, and ALP levels indicates significant differences in disease severity, reflecting this cohort's heterogeneous nature of hepatobiliary dysfunction (Table 2).

**Table 2:** Biochemical Markers in Patients with Suspected Biliary Atresia. Summary of conjugated bilirubin, GGT, and ALP levels, including mean, median, standard deviation, and percentiles. GGT: Gamma-glutamyl transferase

Parameter	N (%)	Mean	Median	Standard Deviation	Minimum	Maximum	25th Percentile	50th Percentile	75th Percentile
Conjugated Bilirubin (µmol/L)	47 (88.7%)	115.3	111.0	63.0	13	310	64	111	149
GGT (IU/L)	46 (86.8%)	405.8	234.0	430.7	31.4	2133	97.8	234	585.8
ALP (IU/L)	51 (96.2%)	626.5	595.0	309.7	21	1713	438	595	769

Table 3 demonstrates that all patients diagnosed with BA had GGT levels above 150 IU/L 21 (100%), whereas only 10 (31.3%) of patients without BA exhibited elevated GGT levels. The mean GGT level was significantly higher in BA patients (550.2 IU/L) compared to non-BA patients (234.5 IU/L). These findings support the use of GGT as a distinguishing biomarker for BA.

**Table 3:** Comparison of GGT Levels in Biliary Atresia (BA) vs. Non-BA Patients. GGT: Gamma-glutamyl transferase

Diagnosis	Patients (N)	Mean GGT (IU/L)	GGT > 150 IU/L (N, %)	GGT < 150 IU/L (N, %)
Biliary Atresia (BA)	21 (39.6%)	550.2	21 (100%)	0 (0%)
No Biliary Atresia (Non-BA)	32 (60.4%)	234.5	10 (31.3%)	22 (68.7%)
Total	53 (100%)	405.8	31 (58.5%)	22 (41.5%)

Ultrasound examination was performed in 53 patients. All scans followed standard sonographic guidelines, including a minimum of four hours of fasting before the procedure. Findings were available for 45 cases (84.9%). The valid percentage distribution showed that 14 (26.4%) of cases had a normal ultrasound, nine (17%) had gallbladder absence, and three (5.7%) showed the triangular cord sign alone, with two (3.8%) displaying both findings. Ultrasound data were missing for eight cases (15.1%) (Table 4).

**Table 4:** Distribution of Ultrasound Findings.

Ultrasound Finding	Number of Cases	Percentage of Total (%)
Normal Ultrasound	14	26.4
Gallbladder Absence	9	17.0
Triangular Cord Sign Alone	3	5.7
Both Findings (Gallbladder Absence + Triangular Cord)	2	3.8
Missing Data	8	15.1
Total Cases Examined	53	100.0

The HIDA scan results were abnormal in 33 cases (62.3%) out of 53 patients, while 20 cases (37.7%) were classified as normal​. The main findings of the abnormal HIDA scan were poor liver washout in 28 patients (84.9%), non-visualization of the gallbladder in 18 patients (54.5%), and no bile excretion into the bowel loops in 14 patients (42.4%). The most frequently reported HIDA diagnoses were BA, with 28 (52.8% of the dataset) patients diagnosed with BA in HIDA reports. In contrast, 25 patients (47.2% of cases) were reported as having no evidence of BA, and eight patients (15%) were identified with neonatal hepatitis (Table 5).

**Table 5:** Summary of HIDA Scan Findings. HIDA scan: Hepatobiliary iminodiacetic acid scan

HIDA Scan Findings	Number of Cases	Percentage (%)
Overall HIDA Result		
Abnormal	33	62.3 (of 53 Patients)
Normal	20	37.7 (of 53 Patients)
Findings Among Abnormal HIDA Scans		
Poor Liver Washout	28	84.9 (of 33 Abnormal Cases)
Non-visualization of the Gallbladder	18	54.5 (of 33 abnormal cases)
No Bile Excretion into the Bowel Loops	14	42.4 (of 33 abnormal cases)
Diagnostic Interpretation (Total Patients = 53)		
Diagnosed with Biliary Atresia	28	52.8
No Evidence of Biliary Atresia	25	47.2
Neonatal Hepatitis	8	15.1

A total of 25 patients (47.2%) underwent a biopsy, while 52.8% did not. Among those who had a biopsy, nine patients (36.0%) were confirmed to have BA based on histopathological analysis, while 16 patients (64.0%) were negative for BA. Additionally, five patients (20.8%) were diagnosed with neonatal hepatitis, highlighting the variety of liver conditions present in this group. Other diagnoses included conditions like Alagille syndrome (three patients), cholestatic hepatitis, cirrhosis, and rare metabolic disorders such as Niemann-Pick disease (Type C): one case, Na+-independent SLC12A6 mutation syndrome (NISCH) syndrome: one case, and Sanjad Sukati syndrome: one case. These syndromes are associated with biliary tract anomalies, usually presenting with cholestatic jaundice.

The HIDA scan and histopathology results were compared using cross-tabulation (Table 6). The results showed that of the nine patients diagnosed with BA via histopathology, all were correctly identified by the HIDA scan (100% sensitivity). However, seven patients who did not have BA based on histopathology were incorrectly diagnosed with BA by the HIDA scan.

**Table 6:** Accuracy of HIDA Scan vs. Histopathologically Confirmed Biliary Atresia. This table compares HIDA scan results with histopathology findings. HIDA scan: Hepatobiliary iminodiacetic acid scan

HIDA Diagnosis	Biliary Atresia Confirmed by Histopathology	No Biliary Atresia Confirmed by Histopathology	Total
Positive HIDA	9 (36%)	9 (36%)	18 (72%)
Negative HIDA	0 (0%)	7 (28%)	7 (28%)
Total	9 (36%)	16 (64%)	25 (100%)

However, only a small number of the patients underwent liver biopsy and around half of the patients did not have histopathological correlation due to different reasons, the final diagnosis of BA in this study was determined not only based on liver biopsy results but also on clinical outcomes (improvement or lack of improvement) over the follow up period that ranged from two years to eight years. That is because a diagnosis of BA will ultimately result in end-stage liver disease if no intervention is done. According to this criterion and based on clinical follow-up, twenty patients were truly positive for BA (Table 7). Twenty-four patients (45.3%) were confirmed negative. There were eight false-positive results (15.1%), where the HIDA scan suggested BA, but the patients did not have the condition, as they showed clinical improvement without any intervention. However, with HIDA scan findings suggestive of BA, these cases were likely related to neonatal hepatitis. Only one case was false-negative (1.9%), where the HIDA scan missed the diagnosis of BA. 

**Table 7:** HIDA Scan Accuracy vs. Histopathology + Clinical Follow-Up. This table compares HIDA scan results with combined histopathology and clinical outcomes. HIDA scan: Hepatobiliary iminodiacetic acid scan

HIDA Scan Results	Biliary Atresia (Histopathology & Clinical Outcome)	No Biliary Atresia (Histopathology & Clinical Outcome)	Total
HIDA Positive	20 (37.7%)	8 (15.1%)	28 (52.8%)
HIDA Negative	1 (1.9%)	24 (45.3%)	25 (47.2%)
Total	21 (39.6%)	32 (60.4%)	53 (100.0%)

Based on the above data, the diagnostic accuracy of the HIDA scan for diagnosing BA is shown in Table 8.

**Table 8:** Diagnostic Accuracy of the HIDA Scan for Biliary Atresia This table presents key diagnostic performance metrics of the HIDA scan, including sensitivity, specificity, predictive values, likelihood ratios, and overall accuracy, with corresponding 95% confidence intervals (CI). HIDA scan: Hepatobiliary iminodiacetic acid scan

Statistic	Value	95% CI
Sensitivity	95.24%	76.18% to 99.88%
Specificity	75.00%	56.60% to 88.54%
Positive Likelihood Ratio	3.81	2.07 to 7.00
Negative Likelihood Ratio	0.06	0.01 to 0.43
Disease Prevalence	39.62%	26.45% to 54.00%
Positive Predictive Value	71.43%	57.65% to 82.11%
Negative Predictive Value	96.00%	77.81% to 99.39%
Accuracy	83.02%	70.20% to 91.93%

## Discussion

Comparing HIDA scan results with final diagnoses and clinical follow-ups in patients with BA provides essential insights into the utility of this diagnostic process. Multiple studies have investigated the role of the HIDA scan in evaluating neonatal jaundice, with a particular focus on its utility in diagnosing BA. However, no such study has been conducted in our tertiary hospital, and we aimed to compare our diagnostic accuracy with that reported in other international publications. Our study demonstrated a sensitivity of 95.2% and a specificity of 75.0% for HIDA scintigraphy in diagnosing BA, aligning with reviews that report hepatobiliary scintigraphy's sensitivity ranging from 97% to 100% and specificity ranging from 33% to 91% [8,10]. However, our study's PPV was 71.4%, notably lower than the 88.0% PPV reported by Chan et al. [8]. This difference may be attributed to our cohort having more false-positive cases. Such overlap is well-documented in conditions like NH, Alagille syndrome, and certain metabolic disorders (e.g., Niemann-Pick disease type C, cystic fibrosis-related liver disease) that can present with similar hepatobiliary dysfunction, leading to delayed biliary excretion and a misinterpretation of HIDA scan results [6]. The overall diagnostic accuracy of our cohort is 83.0% in this study, consistent with the 80-90% range reported in the literature [8,10]. Despite moderate specificity, the high sensitivity and NPV make the HIDA scan a valuable diagnostic tool for BA. One of the key limitations of HIDA scintigraphy in diagnosing BA is its inability to reliably distinguish BA from other causes of neonatal cholestasis, such as NH and metabolic liver disorders. To address this challenge, Jancelewicz et al. (2015) proposed incorporating biochemical markers, such as GGT and conjugated bilirubin levels, to enhance the specificity of BA diagnosis [11]. Their study found that elevated GGT levels (>150 IU/L) were strongly associated with BA, whereas lower levels were more indicative of NH. Similarly, in our study, all patients diagnosed with BA had GGT levels above 150 IU/L (Table 3), reinforcing the reliability of this marker in distinguishing BA from other cholestatic conditions. There is an acknowledgment of variability in the diagnostic accuracy of HIDA scans. Inamullah et al. highlighted that HIDA scans demonstrate significant accuracy (up to 98.1%) when combined with high GGT levels. Misdiagnosis is possible, particularly in cases where NH mimics BA [12].

Because potential HIDA scan diagnoses overlap, some studies emphasized the importance of using HIDA scans as part of a broader diagnostic algorithm rather than as a standalone tool. For instance, Jancelewicz et al. proposed a screening algorithm to exclude BA in infants with neonatal jaundice efficiently. They found that conjugated bilirubin levels of less than 2.5 mg/dL, gamma-glutamyl transpeptidase levels of less than 150 U/L, excretion on HIDA, or a normal percutaneous cholangiogram were 100% sensitive for excluding BA [11]. Their screening algorithm aimed to reduce invasive procedures, such as laparotomy, and, when combined with HIDA results, helped avoid unnecessary surgeries.

HIDA scan findings, complemented by liver biopsy, significantly improved the specificity of BA diagnosis [12]. A liver biopsy provides a more definitive diagnosis, distinguishing between BA and other liver-related conditions. However, some parents refuse the biopsy for their children, like the two cases in this cohort. The delayed biopsy led to delayed management and the subsequent expected result of liver cirrhosis and, ultimately, death in one patient, as he was unfit for a liver transplant.

Other studies have investigated long-term outcomes for patients with BA post-Kasai portoenterostomy and showed improved patients’ prognosis when HIDA scanning provides early and accurate diagnosis [11]. In our cohort, only six patients underwent Kasai portoenterostomy. All of them were accurately diagnosed at the age of less than 60 days by HIDA scan. Chardot also noted that delayed diagnosis contributes to progressive liver damage, making liver transplantation more likely, underscoring the importance of early detection strategies in BA management [13].

Conditions such as choledochal cysts and cystic fibrosis-related liver disease can also present with similar cholestasis patterns, where excretion of the tracer is reduced or absent, complicating the differentiation of BA from these conditions on HIDA scans alone [9].

Despite its high sensitivity, HIDA scanning alone cannot definitively rule out BA without supplemental procedures such as liver biopsy or cholangiography, especially in cases with ambiguous results. The need for multidimensional diagnostic approaches remains crucial, as demonstrated in various studies [9].

Limitations

This study has some limitations. Being retrospective, it relies on medical records, which may introduce bias and inconsistencies. Only 47.2% of patients underwent a liver biopsy, meaning many diagnoses were based on clinical follow-up rather than histopathological findings. The small sample size (n = 53) from a single center also limits generalizability. Despite these challenges, this study highlights the value of HIDA scans in diagnosing BA.

## Conclusions

The HIDA scan is a valuable tool in the diagnostic workup for BA, particularly when combined with other clinical indicators like elevated GGT levels and a lack of gallbladder visualization. However, it should not be used in isolation, and a comprehensive diagnostic strategy, often incorporating liver biopsy and cholangiography, is essential for accurate diagnosis and improved clinical outcomes.

Despite its limitations, HIDA scintigraphy's noninvasive nature makes it a useful initial screening tool. Future advancements in imaging technology and standardized reporting criteria may enhance its reliability. Multicenter studies with larger cohorts may contribute to the optimization of diagnostic algorithms by validating specific imaging criteria and laboratory markers, enhancing targeted management strategies.
